# Assessment of Six Blackberry Cultivars Using a Combination of Metabolomics, Biological Activity, and Network Pharmacology Approaches

**DOI:** 10.3390/antiox13030319

**Published:** 2024-03-06

**Authors:** Hana Lee, Zhixin Wang, Zhanao Deng, Yu Wang

**Affiliations:** 1 Department of Food Science and Human Nutrition, Citrus Research and Education Center, University of Florida, Lake Alfred, FL 33850, USA; lee.hana@ufl.edu (H.L.); zhixin.wang@ufl.edu (Z.W.); 2 Department of Environmental Horticulture, Gulf Coast Research and Education Center, University of Florida, Institute of Food and Agricultural Sciences, Wimauma, FL 33598, USA; zdeng@ufl.edu

**Keywords:** blackberry, metabolomics, antioxidant, anti-inflammation, network pharmacology

## Abstract

Blackberries have gained considerable attention due to their high antioxidant content and potential health benefits. This study compared the metabolite profiles of six blackberry cultivars and investigated their biological activities. The metabolites extracted from blackberries were analyzed using metabolomics, and their biological activities and mechanisms were confirmed using in vitro models and network pharmacology. Among the cultivars examined, “Kiowa” ripe berries exhibited the highest antioxidant and anti-inflammatory activities. These effects were primarily attributed to the accumulation of flavonoids (quercitrin and luteolin) and anthocyanin (cyanidin 3-*O*-glucoside) in the phenylpropanoid pathway. Furthermore, our research identified 13 blackberry metabolites that interacted with 31 genes, including AKT1, CASP3, JUN, MAPK8, NOS3, NQO1, and HMOX1 which play roles in reducing oxidative stress, protecting cells from damage, and suppressing inflammation. These findings suggest that blackberry metabolites, such as quercitrin, luteolin, and cyanidin 3-*O*-glucoside, may exert therapeutic effects by modulating specific genes and pathways associated with antioxidant and anti-inflammatory responses. This research is promising not only for plant breeders but also for those interested in harnessing the health-promoting properties of blackberries.

## 1. Introduction

Scientific investigations have unequivocally established the manifold health benefits of incorporating fruits and vegetables into human diet [1]. Among the numerous bioactive compounds found in these nutritional powerhouses, flavonoids, a type of polyphenolic compounds, have gained prominence due to their roles in conferring antioxidant, anti-inflammatory, and anticancer properties [2]. The inclusion of fruits and vegetables in the daily dietary practices has garnered strong recommendations from a plethora of studies. Notably, berries, which ranked among the top 50 antioxidant-rich foods, are particularly encouraged [3].

Blackberries, a delectable fruit produced by plant species within the genus *Rubus* in the family *Rosaceae* [4], have attracted attention due to their substantial nutritional values. The commercial production of blackberries is estimated to be approximately 154,578 tons annually in North America, Europe, Asia, South America, Oceania, Central America, and Africa [5]. Blackberry cultivation has rapidly increased because of the introduction of new and improved cultivars and consumers’ interest in its high nutritional and medicinal value [6,7]. Blackberry is a rich source of phenolic compounds such as anthocyanins, flavan 3-ols, and phenolic acids [4]. In particular, cyanidin 3-*O*-glucoside is consistently the predominant anthocyanin, which is responsible for the dark color of blackberries and has been reported to exert prominent bioactivity, including anti-inflammatory, antioxidant, anti-cancer, and anti-neurodegenerative activities [8,9]. A previous study reported that the antioxidant capacity of blackberries was higher than those of blueberries, raspberries, and cherries [10]. Moreover, blackberries have a higher inhibitory activity against free radicals than cranberries and strawberries [11]. The demand for high-quality blackberries has led to the development of numerous cultivars with varying levels of bioactive compounds and antioxidant capacities. Considering the rapid expansion of applications using various blackberry cultivars and the importance of their efficient use, it is essential to understand their nutritional profiles. Comparing different blackberry cultivars on the basis of their metabolite profiles and biological activities can help consumers, food processors, and health professionals in selecting the most appropriate cultivars for their specific needs. In addition, breeders can focus on selecting cultivars with the desired traits and genetic markers, thereby accelerating the development of blackberry varieties that satisfy both consumer preferences and industry demands.

In recent years, targeted and untargeted metabolomics approaches have emerged as powerful tools for the comprehensive analysis of the metabolic profiles of different plant species [12]. In general, targeted analysis aims to identify and quantify a limited number of known compounds using liquid chromatography–mass spectrometry or gas chromatography–mass spectrometry [13]. Untargeted analysis focuses on acquiring data for as many species of metabolites as possible, annotating them, and reviewing both known and unknown metabolic changes using principal component analysis (PCA) and partial least-squares-discriminant analysis (PLS-DA) [14]. The results can be used to make relative comparisons among sample groups and provide hypotheses that can be further investigated using targeted approaches. Several studies have used metabolomics to compare the metabolic profiles of blackberries [15,16]. These studies have identified differences in the levels of various metabolites, including sugars, organic acids, flavonoids, and anthocyanins [17]. A previous study investigated the levels of sugar and organic acid among 26 blackberry cultivars and found significant differences [18]. Some cultivars contained higher levels of sucrose and organic acid than others. Another study found that the levels of anthocyanins, which are responsible for the red, purple, and blue pigments in blackberries, varied widely among 11 blackberry cultivars, with certain cultivars possessing higher levels than the others [19]. These metabolic differences can affect the quality and health benefits of blackberries. For example, anthocyanin levels are typically linked to increased antioxidant potential, which may offer health advantages, including a reduced risk of cancer and cardiovascular diseases [20].

Network pharmacology is an advantageous approach for understanding the cellular mechanisms underlying specific diseases and uncovering potential targets and pathways [21]. In recent years, there has been a successful application of network pharmacology in constructing and illustrating networks involving diseases, genes, and targeted drugs [22]. Considering that foods consist of multiple compounds, and the network pharmacology approach focuses on the concept of “network target, multicomponent therapeutics”, similar to the holistic nature of complex matrices found in medicinal herbs, this strategy is deemed suitable for comprehending the mechanisms of action of diverse food materials [23]. In traditional research, the study of biological activity has been focused on identifying a single bioactive compound in each extract [24,25,26]. In this study, metabolic profiling, biological activity assessment, and network pharmacology approach were integrated to investigate the relationship between various metabolites and biological activities instead of identifying biological activities of a single compound. Once the compounds influencing biological activities are clearly identified and validated, the genes regulating the biosynthesis of these compounds can be found. This will enable the development of selection tools that empower breeders to modulate bioactive compounds more effectively. This study aimed to compare the metabolite profiles of six distinct blackberry cultivars and contribute to a deeper understanding of the relationship between blackberry metabolites and biological activities/mechanisms.

## 2. Materials and Methods

### 2.1. Materials

Acetonitrile, water (LC-MS grade), dimethyl sulfoxide, 2,2-azino-bis-3-ethylbenzothiazoline-6-sulfonic acid (ABTS), 2′7′-dichlorofluorescein diacetate (DCFH-DA), dibutyl hydroxy toluene, 1,1-diphenyl-2-picrylhydrazyl (DPPH), ferric chloride, ferrous sulfate, aluminum trichloride (AlCl_3_), Folin–Ciocalteu reagent, and sodium carbonate were purchased from VWR International (Suwanee, GA, USA). Phosphate buffered saline (PBS), Dulbecco’s modified eagle medium (DMEM), and fetal bovine serum (FBS) were obtained from Thermo Fisher Scientific (Waltham, MA, USA).

### 2.2. Plant Materials

Two commercial blackberry cultivars (“Caddo” and “Kiowa”) and three experimental cultivars (BL4, BL5, and BL7) grown at the University of Florida Gulf Coast Research and Education Center (GCREC) in Wimauma, FL, USA were selected for this study. In addition, the commercial blackberry (CM), “Navaho”, was purchased from the local Publix Super Markets. Blackberries at the GCREC research orchard were produced using a horticultural system that is commonly employed for blackberry production—plants were planted on raised mulched beds, supported on metal posts, irrigated with an automated drip irrigation system, and fertilized with liquid fertilizers through the drip tubes. Fully ripe blackberries that were shiny black with a plump, full, and slightly tender feel were collected manually on 18 May and again on 24 May 2022 (i.e., two harvests). The harvested berries were kept inside a cooler (approximately 10 °C) with ice packs for about 4 h before they were frozen. Standard industry practices were followed to handle the harvested berries, and they were not subject to additional washing or rinsing, similar to the purchased berry samples. All samples were flash frozen in liquid nitrogen and stored at −80 °C until processing. For each cultivar, three biological replicates were from three different field plots, with five plants per plot.

### 2.3. Sample Preparation

Blackberry samples were ground into fine powder under the protection of liquid nitrogen. Metabolites were extracted from frozen berry powder using 50% methanol. Genistein-*d*_4_, citric acid-2,2,4,4-*d*_4_, D-sorbitol-^13^C_6_, D-glucose-^13^C_6_, salicylic acid-*d*_4_, proline 2,5,5-*d*_3_, and hippuric acid-d_5_ were used as internal standards. Samples were prepared according to a previously described method [27]. To determine antioxidant content and anti-inflammatory effects, blackberries were extracted using 50% methanol without internal standards.

### 2.4. Untargeted Analysis Using LC-MS/MS

Chromatographic separation was performed on a Vanquish Flex Binary UHPLC (Thermo Fisher Scientific, Waltham, MA, USA). An Agilent InfinityLab Poroshell HPH-C_18_ column (150 × 2.1 mm, 1.9 μm) and an Agilent InfinityLab Poroshell 120 HILIC-Z (150 × 2.1 mm, 2.7 μm) column were used for separation. The column temperature was maintained at 40 °C. For the reversed phased C_18_ (RP-C_18_) separation system, the mobile phase was composed of water with 0.1% formic acid (phase A) and acetonitrile with 0.1% formic acid (phase B), and the gradient elution program was as follows: 0–28 min, 2–42% B; 28–32 min, 42–100% B; 32–33 min, 100–2% B; 33–37 min, 2% B. For the hydrophilic interaction liquid chromatography (HILIC) separation system, the mobile phase was composed of 10 mM ammonium acetate (pH 9.0) in water (phase A) and 10 mM ammonium acetate (pH 9.0) in water/acetonitrile 10:90 *v*/*v* (phase B), and the elution program was as follows: 0–20 min, 100–75% B; 20–22 min, 75–5% B; 22–25 min, 5% B; 25–27 min, 5–100% B; 27–37 min, 100% B. The flow rate was set at 0.4 mL/min. The conditions of the Q Exactive Plus Orbitrap mass spectrometer system (Thermo Fisher Scientific, Waltham, MA, USA) coupled with the UHPLC were described in a previous publication [27].

### 2.5. Targeted Analysis Using LC-MS/MS

The flavonoids and phenolic acids were analyzed using an Ultimate 3000 UHPLC system coupled to a TSQ Quantiva triple quadrupole mass spectrometer (Thermo Fisher Scientific, San Jose, CA, USA). The column temperature was maintained at 30 °C and the flow rate was set at 0.3 mL/min. The flavonoids and phenolic acids were separated on an Agilent InfinityLab Poroshell HPH-C_18_ column (150 × 2.1 mm, 1.9 μm) using 0.1% formic acid in water (phase A) and 0.1% formic acid in acetonitrile (phase B). The gradient elution program was as follows: 0–4 min, 20–40% B; 4–5.5 min, 40–90% B; 5.5–8 min, 90% B; 8–8 min, 90–20% B; 8–10 min, 20% B. The optimum values for each scan, including retention time, polarity, precursor–product ion pairs, collision energies, and RF lens, are listed in Table 1. Flavonoids and phenolic acids were quantified using isotope-labeled internal standards (genistein-*d*_4_ and salicylic acid-*d*_4_) based on the peak ratio and concentration of the internal standards. The optimal scan parameters for the targeted compounds are shown in Supplementary Table S2.

### 2.6. Analysis Based on Network Pharmacology

The compounds identified through untargeted and targeted analyses were represented in the SMILES format. Target genes associated with the selected compounds were identified using the STITCH database ver. 5.0 (http://stitch.embl.de/; accessed on 11 November 2023). For functional annotation and the exploration of signaling pathways, biological processes, cellular components, and molecular functions closely related to anti-inflammatory and antioxidant target proteins, DAVID ver. 6.8 (https://david.ncifcrf.gov/; accessed on 18 November 2023) and Kyoto Encyclopedia of Genes and Genomes (KEGG) pathways (http://www.genome.jp/kegg/pathway.html; accessed on 18 November 2023) were used. The protein–protein interaction network (PPI network) was constructed using the STRING database (https://string-db.org/; accessed on 21 November 2023). The gene-pathway network was visualized using Cytoscape ver. 3.10.

### 2.7. Determination of Total Phenolic, Flavonoid, and Anthocyanin Contents and Antioxidant Activities

The total phenolic content (TPC) of blackberries was measured using the Folin–Ciocalteu colorimetric method [28]. The total flavonoid content (TFC) was confirmed using a colorimetric method described in a previous study [29]. TPC and TFC were expressed as gallic acid equivalent and catechin equivalent, respectively. The total anthocyanin content was determined using the pH differential method [28]. The results were expressed as cyanidin 3-*O*-glucoside equivalent. DPPH and ABTS radical scavenging activities and reducing power were determined according to a previously described method [30]. DPPH and ABTS radical scavenging activities were expressed as Trolox equivalents.

### 2.8. Cell Culture and Cell Viability

RAW 264.7 cells were derived from mouse macrophage and were acquired from ATCC (Manassas, VA, USA). These cells were cultivated in a humidified environment of 5% CO_2_ at 37 °C using 10% FBS/DMEM supplemented with 100 U/mL penicillin and 100 mg/mL streptomycin. Cell viability was assessed using the MTT assay. The cells were seeded into 96-well plates at a density of 1.5 × 10^5^ cells/well and incubated for 24 h before replacing the culture medium with serum-free medium containing blackberry extracts. The cells were then incubated for 24 h before adding the MTT reagent (1 mg/mL) to each well, followed by a 2 h incubation period. The medium was then removed, and DMSO was used to dissolve the blue crystallized formazans. The absorbance at 550 nm was measured using a FlexStation 3 Multi-Mode Microplate Reader (Molecular Devices, Silicon Valley, CA, USA).

### 2.9. Measurement of Nitric Oxide (NO), Tumor Necrosis Factor-α (TNF-α), Protective Effect, and Intercellular Reactive Oxygen Species (ROS) Levels

To confirm nitric oxide production, the cell culture supernatant was mixed with Griess reagent, and absorbance was recorded using a microplate reader. To determine the nitrite concentration, the mixture’s absorbance was compared to sodium nitrite dilutions that served as a standard. Additionally, Invitrogen™ TNF alpha Mouse ELISA kits (Invitrogen, Carlsbad, CA, USA) was used to detect and quantify mouse TNF-α concentrations in the culture supernatants, following the manufacturer’s instructions. The protective effect was measured using an MTT assay. The ROS generation level was confirmed by measuring fluorescence intensity with DCFH-DA, and a microplate reader was used to analyze this intensity over a 2 h period.

### 2.10. Data Processing and Statistical Analysis

To analyze the untargeted metabolomics data, an empirical workflow was performed using Compound Discoverer v3.3 (Thermo Fisher Scientific, Waltham, MA, USA). This involved aligning retention times, detecting and grouping peaks, deducing molecular formulas, and searching a database for identification. A systematic strategy was used to annotate compounds. First, a formula predictor was used to determine the elemental composition of each peak based on the protonated molecular ion and isobaric ions, with a maximum mass accuracy error of ±3 ppm. Then, known compounds were recognized by matching them with the in-house KEGG pathway metabolites database. The annotations were manually confirmed by MS/MS fragmentation patterns. As for the targeted metabolomics, the data processing was performed using Xcalibur 4.0 (Thermo Fisher Scientific, Waltham, MA, USA). The raw data were normalized by mean centering and dividing by the standard deviation of each variable. MetaboAnalyst (https://www.metaboanalyst.ca; accessed on 7 October 2023) was used for PLS-DA. Cytoscape ver. 3.10. was used for network visualization. Pearson correlation analysis was performed using PASW Statistics 18.

## 3. Results and Discussion

### 3.1. Metabolite Profiling of Six Blackberry Cultivars

**Figure 1:**
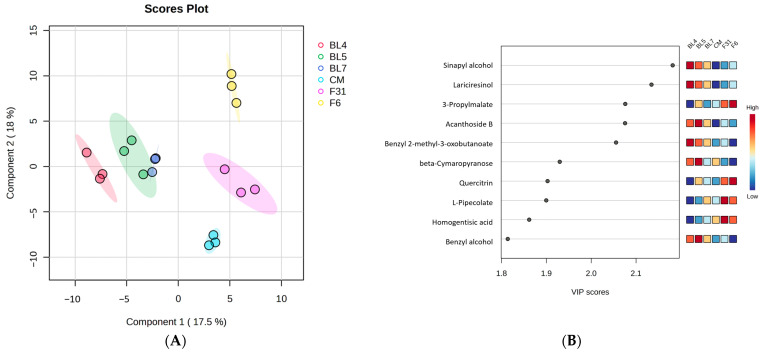

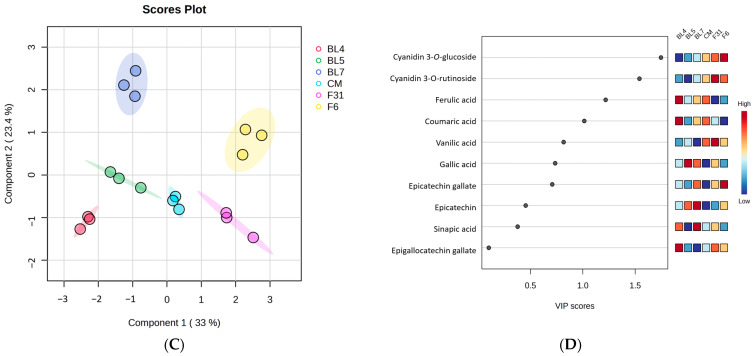
PLS-DA scores plot and VIP scores from untargeted metabolomics (**A**,**B**) and targeted metabolomics (**C**,**D**) of ripe berries from six blackberry cultivars (F31 = “Caddo”, and F6 = “Kiowa”).

Metabolomics analysis using LC/MS has been applied to various studies of fruit, such as cultivar selection, phytochemical analysis, metabolic regulation, and stress response [31,32,33]. In this study, we conducted untargeted metabolomics analysis on six different blackberry cultivars to explore their comprehensive metabolic profiles. The features were tentatively annotated on the basis of the MS^2^ fragmentation patterns and MS database. A total of 178 metabolites (Supplementary Table S1) were screened by PLS-DA with a model prescribed by component 1/component 2, accounting for 35.5% of the variance in compositional makeup among the different blackberry cultivars (Figure 1A). “Caddo”, “Kiowa”, and CM blackberries were clearly separated from the three BL experimental cultivars. As shown in Figure 1B, sinapyl alcohol and lariciresinol had high variable importance in projection (VIP) scores (2.18 and 2.13, respectively) among the top 15 metabolites (VIP > 1.0). “Caddo” and “Kiowa” cultivars showed the highest levels of 3-propylmalate and quercitrin, which also indicated relatively high VIP scores.

We noticed that some compounds known to be abundant in blackberries, such as anthocyanins and flavan-3-ols, were not detected by untargeted metabolomics analysis. These results emphasize the limitations of untargeted metabolomics in analyzing specific compounds of interest. To confirm these flavonoids and phenolic acids, we performed a targeted analysis. The contents of flavonoids and phenolic acids in the analyzed blackberries are shown in Table 1. Significant differences were found in the flavonoid composition of six blackberry cultivars. The level of cyanidin-3-*O*-glucoside was between 1201.7–3530.8 µg/g DW and the level of cyanidin-3-*O*-rutinoside was between 4.3–620.5 µg/g DW. “Kiowa” and “Caddo” cultivars were characterized by the highest amount of cyanidin-3-*O*-glucoside and cyanidin-3-*O*-rutinoside, respectively. Cyanidin-3-*O*-glucoside is the chief anthocyanin, comprising 92% of all anthocyanins in blackberries [34]. A previous study has shown that concentrations of cyanidin-3-*O*-glucoside and cyanidin-3-*O*-rutinoside in “Thornless Evergreen” cultivar were 405.1 and 29.3 µg/g fresh weight, respectively [34]. These differences in the content are believed to be due to variations in blackberry varieties and moisture contents. The beneficial health effects of blackberries may be due to the bioactivity of anthocyanins. The cultivars BL4 and BL5 were characterized by the highest epicatechin concentration. Epicatechin is consistently the most abundant flavan-3-ol in blackberry and has been reported at concentrations of 448.4 μg/100 g DW [35,36]. The level of epicatechin gallate was between 0.021–0.256 µg/g DW and the level of epigallocatechin gallate was between 0.072–0.342 µg/g DW. There were roughly similar amounts of gallic acid, vanillic acid, coumaric acid, ferulic acid, and sinapic acid in these blackberry cultivars. In previous studies, a low amount of phenolic acids including ferulic acid (2.99–3.51 mg/100 g fresh weight), coumaric acid (0.40–2.08 mg/100 g fresh weight), caffeic acid (1.38–3.64 mg/100 g fresh weight), and gallic acid (3.4–6.4 mg/100 g fresh weight) was detected in blackberries [37,38]. The anthocyanin content of blackberries was higher than that of phenolic acids and flavan-3-ols, which implies that anthocyanins are the most important subclass of phenols among compounds individually identified.

Our analysis highlights the inherent limitations of untargeted metabolomics, particularly in detecting specific compounds of interest such as anthocyanins and flavan-3-ols, which are known to be abundant in blackberries. To overcome this, targeted analysis was employed, confirming the presence and quantifying the levels of these flavonoids and phenolic acids. This approach underlines the significance of combining both untargeted and targeted methods for a comprehensive metabolite profiling.

Our findings contribute valuable insights into the metabolite composition of different blackberry cultivars, particularly in terms of their flavonoid and phenolic acid content. These findings are crucial for the selective breeding of blackberry varieties, enabling the development of cultivars with enhanced nutritional and functional attributes, tailored to meet specific health benefits and consumer preferences.

**Table 1:** Flavonoid and phenolic acid contents of blackberries.

	BL4	BL5	BL7	F31 (Caddo)	F6 (Kiowa)	CM (Navaho)
Flavonoid content (µg/g dry weight)						
Cyanidin-3-glucoside	1201.7 ± 47.4 ^e^	1688.1 ± 171.5 ^d^	2245.6 ± 80.5 ^c^	2672.5 ± 197.7 ^b^	3530.8 ± 197.8 ^a^	2595.2 ± 71.5 ^b^
Cyanidin-3-rutinoside	5.8 ± 0.3 ^c^	4.3 ± 0.2 ^c^	9.3 ± 1.1 ^c^	620.5 ± 43.2 ^a^	444.7 ± 104.5 ^b^	397.1 ± 31.3 ^b^
Epicatechin	551.6 ± 119.3 ^b^	1006.2 ± 35.4 ^a^	1041.2 ± 75.6 ^a^	419.3 ± 117.2 ^b^	713.8 ± 409.3 ^ab^	28.8 ± 3.0 ^c^
Epicatechin gallate	0.114 ± 0.010 ^c^	0.106 ± 0.025 ^c^	0.162 ± 0.030 ^b^	0.115 ± 0.019 ^c^	0.253 ± 0.042 ^a^	0.021 ± 0.001 ^d^
Epigallocatechin gallate	0.342 ± 0.037 ^a^	0.103 ± 0.025 ^c^	0.072 ± 0.011 ^c^	0.335 ± 0.019 ^a^	0.203 ± 0.070 ^b^	0.193 ± 0.017 ^b^
Phenolic acid content (µg/g dry weight)						
Gallic acid	0.012 ± 0.003 ^b^	0.029 ± 0.015 ^a^	0.019 ± 0.008 ^ab^	0.013 ± 0.004 ^b^	0.007 ± 0.003 ^b^	0.006 ± 0.001 ^b^
Vanillic acid	0.014 ± 0.005 ^b^	0.017 ± 0.008 ^b^	0.010 ± 0.004 ^b^	0.047 ± 0.026 ^a^	0.021 ± 0.006 ^b^	0.044 ± 0.005 ^a^
Coumaric acid	0.057 ± 0.005 ^a^	0.021 ± 0.004 ^c^	0.027 ± 0.005 ^c^	0.024 ± 0.004 ^c^	0.020 ± 0.006 ^c^	0.042 ± 0.005 ^b^
Ferulic acid	0.060 ± 0.002 ^a^	0.041 ± 0.012 ^b^	0.042 ± 0.005 ^b^	0.026 ± 0.004 ^c^	0.028 ± 0.009 ^c^	0.057 ± 0.002 ^a^
Sinapic acid	0.029 ± 0.004 ^b^	0.018 ± 0.004 ^c^	0.057 ± 0.006 ^a^	0.027 ± 0.006 ^bc^	0.019 ± 0.006 ^c^	0.022 ± 0.003 ^bc^

Different letters in the same row indicate significant differences according to Duncan’s test (*p* < 0.05).

### 3.2. Antioxidant Contents and Activities of Six Blackberry Cultivars

**Figure 2:**
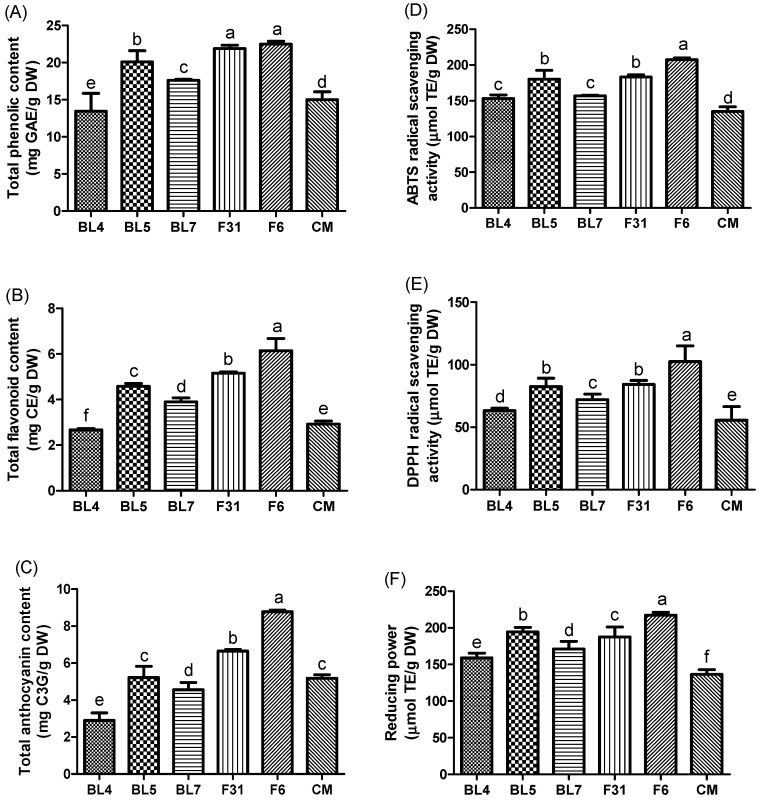
(**A**) TPC, (**B**) TFC, (**C**) TAC, (**D**) ABTS, (**E**) DPPH, and (**F**) reducing power of the six blackberry cultivars (F31 = “Caddo”, F6 = “Kiowa”, CM = “Navaho” from a commercial market). Each value was expressed as the mean values ± standard error (*n* = 3). The different lowercase letters represent significant differences according to Duncan’s multiple-range test (*p* < 0.05). Total polyphenolic content is expressed as gallic acid equivalent mg/g dry weight. Total flavonoid content is expressed as catechin equivalent to mg/g dry weight. Total anthocyanin content is expressed as cyanidin-3-glucoside equivalent to mg/g dry weight. ABTS radical scavenging activity is expressed as trolox equivalent mg/g dry weight, and DPPH radical scavenging activity is expressed as trolox equivalent mg/g dry weight. Reducing power is expressed as trolox equivalent mg/g dry weight.

Blackberries have attracted attention for their abundant phytochemicals and health benefits [35]. To compare the antioxidant contents and activities of six blackberry cultivars, we measured total phenolic content (TPC), total flavonoid content (TFC), and total anthocyanin contents (TAC) and antioxidant activities (Figure 2). The TPC of the blackberry extracts was 13.45–22.49 mg gallic acid equivalent/g DW; TFC of the blackberry extracts was 2.67–6.14 mg catechin equivalent/g DW; TAC of the blackberry extracts was 2.91–8.78 mg cyanidin-3-*O*-glucoside equivalent/g DW (Figure 2A–C). “Kiowa” cultivar contained more than three times TAC compared to the BL4 cultivar. The ABTS and DPPH radical scavenging activities, as well as the reducing power, were in the range of 135.10–207.46, 55.51–102.58, and 136.54–217.10 µM trolox equivalent/g DW, respectively (Figure 2D–F). Previous studies have investigated the TPC in commercial blackberries, including “Triple Crown”, “Thornless”, and “Choktaw”, which ranged from 12.1 to 23.5 mg gallic acid equivalent/g DW [39,40,41]. In another study, the TAC of the blackberry cultivar “Tupy” was estimated to be 3.40 mg cyanidin-3-*O*-glucoside equivalent/g DW, which was comparable to that of the BL7 cultivar (4.56 mg cyanidin-3-*O*-glucoside equivalent/g DW) evaluated in our study [42]. A recent study reported that the ABTS and DPPH radical scavenging activities and reducing power of blackberries were 178.35, 156.95, and 111.63 µM trolox equivalent/g DW, respectively, which is similar to our results [43]. These differences in antioxidant content and activity, depending on cultivars, may be attributed to the varying phytochemical compositions of the six blackberry cultivars. In our study, the CM cultivar (“Navaho”) did not exhibit higher levels of TPC, TAC, ABTS, and DPPH than the other cultivars, as previously reported in another study [44]. Among the six blackberry extracts, “Kiowa” extract showed the highest antioxidant activity, followed by the “Caddo” and BL5 blackberry extracts. The high antioxidant capacity of “Kiowa”, observed in all assays, as compared to the other five blackberry cultivars, could be attributed to its high TPC, TFC, and TAC levels, specifically luteolin, quercitrin, and cyanidin-3-*O*-glucoside. Hence, our results demonstrate the potential of “Kiowa” blackberry cultivar, with its high levels of antioxidant components and activities, as a candidate material for future functional foods.

### 3.3. Anti-Inflammatory Activity of Six Blackberry Cultivars

**Figure 3:**
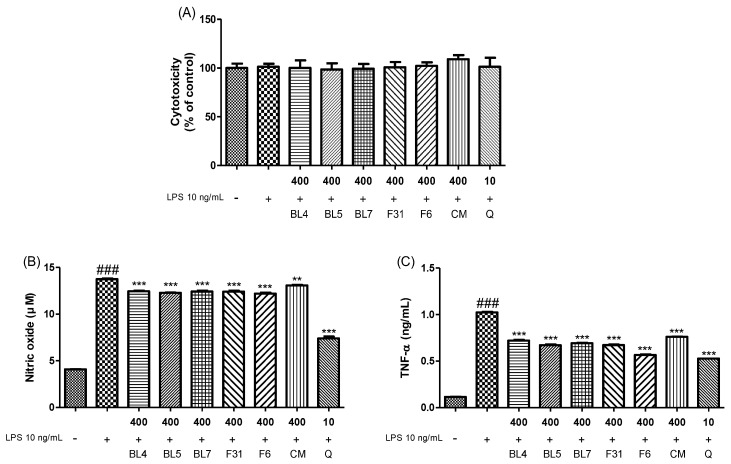

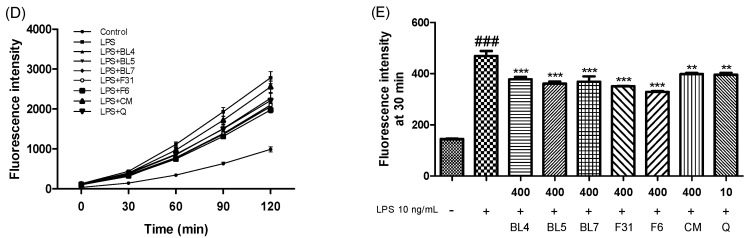
Effect of ripe fruit extract from six blackberry cultivars (F31 = “Caddo”, F6 = “Kiowa”, CM = “Navaho” from a commercial market) on (**A**) cytotoxicity, (**B**) nitric oxide production, (**C**) TNF-α generation, and (**D**,**E**) ROS formation. Each value was expressed as the mean values ± standard error (*n* = 3). ### *p* < 0.001 versus non-treated cells. ** *p* < 0.01 and *** *p* < 0.001 versus LPS treated cells. Blackberry extracts (400 μg/mL) and quercetin (10 μM) were used for cell analysis.

Excessive ROS and NO levels can lead to oxidative stress, which is associated with the development of inflammation-related diseases [45]. Antioxidants derived from natural products protect cells against inflammatory conditions and help maintain the balance of the redox state in the body [46]. Polyphenols, which are abundant in fruits, vegetables, and teas, suppress the production of pro-inflammatory cytokines, such as TNF-α [47]. Blackberries may provide a preventive effect to minimize oxidation and maintain the critical ratio of pro- and anti-inflammatory cytokines and other redox parameters because of its high antioxidant activities [35]. In this study, we compared the protective effect of six blackberry extracts in RAW 264.7 macrophages. Treatment with six blackberry extracts at 400 µg/mL did not result in the cytotoxicity of macrophages (Figure 3A). LPS treatment revealed significant increases in inflammatory mediators such NO and TNF-α in the medium (Figure 3B,C). However, treatment with “Kiowa” blackberry extract markedly reduced the production of the inflammatory mediators. We also confirmed ROS generation in response to treatment with blackberry extracts. Our findings indicate that six blackberry extracts reduced ROS levels by 19.4% (BL4), 23.0% (BL5), 21.4% (BL7), 25.2% (F31), 29.9% (F6), and 15.0% (CM) compared with LPS-treated cells (Figure 3D,E). Our results showed that “Kiowa” blackberry extract was the most effective in reversing the increase in NO, TNF-α, and ROS production. A previous study reported that epicatechin gallate reduced TNF-α-induced activation of NF-κB and consequently secretion of pro-inflammatory cytokine [24]. Quercitrin exerts anti-inflammatory activity by reducing TNF-α production [26]. The anthocyanins present in blackberries are natural antioxidants with strong free radical scavenging capacity and exert anti-inflammatory activities by reducing the release of pro-inflammatory factors and suppressing the activation of the MAPK signaling pathway [20,48]. Furthermore, cyanidin 3-glucoside has been reported to significantly inhibit p-p65 and IkBα degradation levels by suppressing the NF-κB signaling pathway [49]. In addition to anthocyanins, blackberries also contain other polyphenols such as syringin, taxifolin, and luteolin, which have been shown to possess anti-inflammatory activities [26,50,51,52]. The “Kiowa” blackberry extract exhibited significantly higher biological activity than extracts from other blackberry cultivars, suggesting its potential to play a pivotal role in the development of functional foods and medicinal applications involving blackberries.

### 3.4. Correlation between Blackberry Metabolites and Biological Activities and Significant Pathway of Metabolites

**Figure 4:**
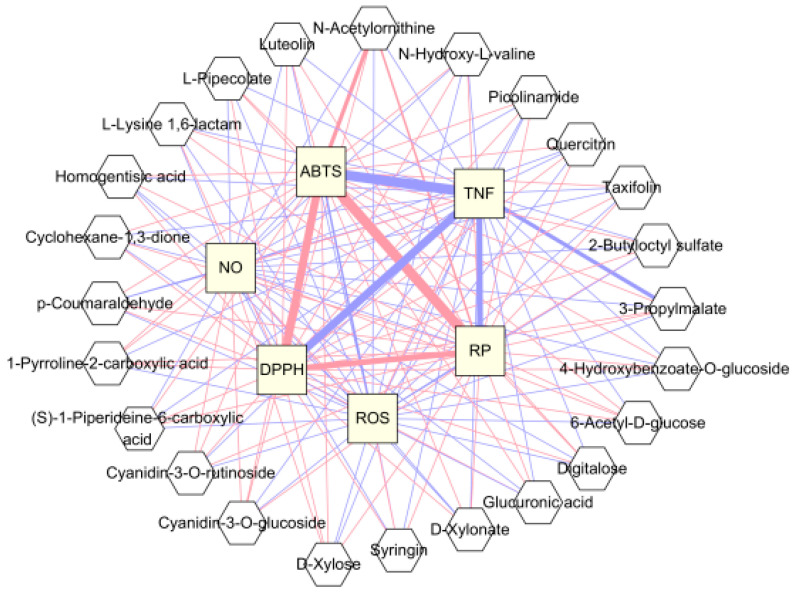
Correlation network visualization between blackberry metabolites and biological activities. A pink edge represents a positive correlation and a blue edge represents a negative correlation. The thicker the edge, the stronger the correlation. ABTS, 2,2′-azino-bis (3-ethylbenzothiazoline-6-sulfonic acid radical scavenging activity; DPPH, 2,2-diphenyl-1-picrylhydrazyl radical scavenging activity; RP, reducing power; NO, nitric oxide; TNF, tumor necrosis factor-α; ROS, reactive oxygen species.

Our findings show that “Kiowa” blackberry extract exhibits excellent antioxidant and anti-inflammatory properties. To investigate which metabolites may influence these characteristics, a correlation network was constructed to analyze the biological activity-related compounds. Among the blackberry metabolites with VIP > 1.0, the correlation of 24 compounds was visualized in Figure 4. These correlations include compounds that exhibit a positive correlation with antioxidant activity and compounds that show a negative correlation with anti-inflammatory activity. Positive correlations of relatively high significance (*p* < 0.01) were found between antioxidant activities and N-acetylornithine, 3-propylmalate, quercitrin, syringin, and luteolin. Significant negative correlations (*p* < 0.01) were observed between TNF-α production and the following metabolites: N-acetylornithine, 3-propylmalate, and quercitrin. In addition, quercitrin was indicated to have a significant negative correlation with ROS level (*p* < 0.01). Previous studies reported that quercitrin directly participates in ROS scavenging and possesses anti-inflammatory activities by suppressing TNF-α [53,54]. Zhao et al. (2023) showed that syringin administration significantly decreased the pro-inflammatory cytokines and ROS levels in animal and cell models [52]. Luteolin effectively alleviated the LPS-induced TNF-α release in serum and exerted the ABTS and DPPH radical scavenging activities in vitro [55,56].

**Figure 5:**
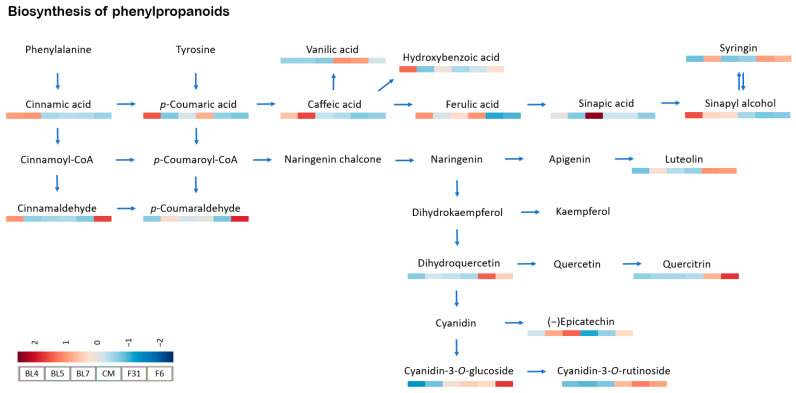
Metabolite mapping on the phenylpropanoids biosynthesis pathway for ripe blackberries.

To determine the significant pathway in blackberry metabolites, all metabolites from untargeted and targeted analyses were used for potential pathway selection by enrichment analysis. The biosynthesis of the phenylpropanoids pathway showed a high number of matched/total metabolites, along with low adjusted *p*-values (3.96 × 10^−7^) and false discovery rate values (4.20 × 10^−5^). A simplified version of the phenylpropanoids biosynthesis pathway was presented for clarity in Figure 5. Among the identified blackberry metabolites, 17 metabolites were recognized in the KEGG database. “Kiowa” blackberry exhibited higher concentrations of cyanidin-3-*O*-glucoside, cyanidin-3-*O*-rutinoside, quercitrin, luteolin, and dihydroquercetin (taxifolin) compared to the other blackberry cultivars. This suggests that “Kiowa” blackberry cultivar may harbor genetic traits enabling it to produce higher quantities of flavonoids or employ efficient mechanisms for their accumulation. Previous studies have well documented that the upregulation of specific genes can lead to the activation and accumulation of flavonoids in plants. Several genes, such as PAL (phenylalanine ammonia lyase) and TAL (tyrosine ammonia lyase), are responsible for the activation of the phenylpropanoid pathway for phenolic biosynthesis [57]. Recent studies have reported that the increased activity of PAL and TAL enzymes in plant materials not only leads to the accumulation of flavonoids but also enhances the protective effects on various cells [58,59,60]. Therefore, the higher levels of flavonoids in “Kiowa” blackberry cultivar suggest that it may exhibit stronger biological effects than the other blackberry varieties. Further research into the underlying genetic mechanisms responsible for these differences may yield valuable applications in breeding and cultivation practices to enhance the nutritional and functional qualities of blackberries. Meanwhile, our study provides valuable insights into the correlation between metabolites and biological activity in six tested blackberry varieties. However, the small sample size limits the generalizability of our results. Future studies should aim to address this limitation by testing a larger and more diverse range of blackberry varieties to obtain more reliable and comprehensive results.

### 3.5. Identification of Target Proteins of Selected Blackberry Metabolites Using Network Pharmacology

**Figure 6:**
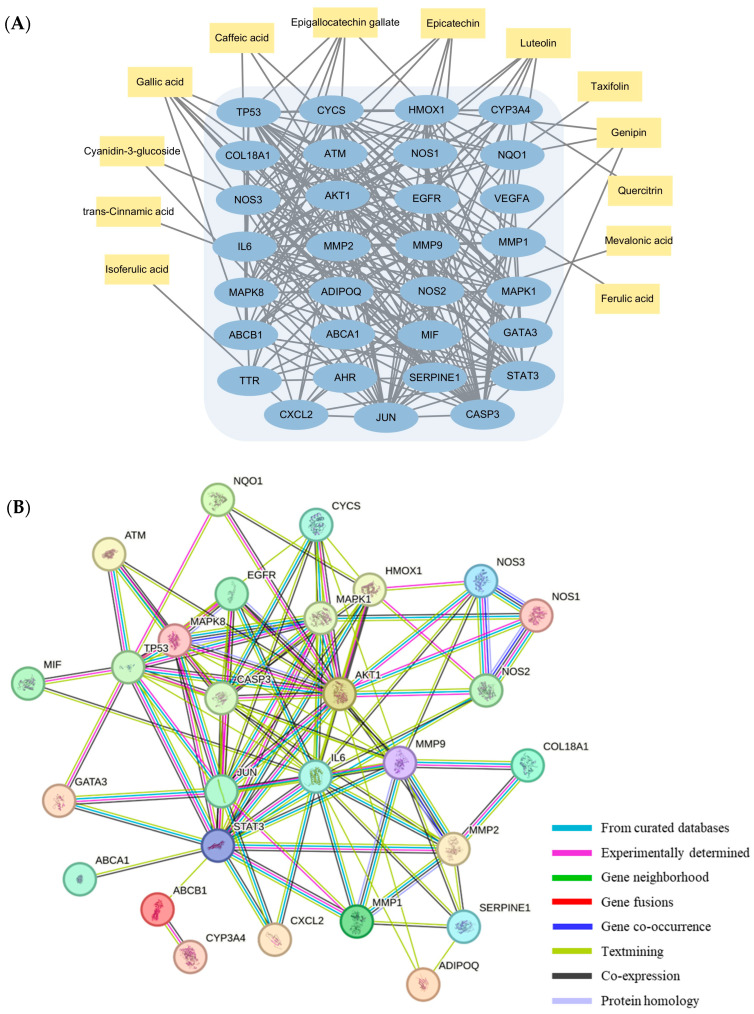
(**A**) Blackberry metabolite–target interaction network. The blue circle represents the target protein of blackberry metabolites. (**B**) Protein–protein interaction (PPI) network of identified anti-inflammation and antioxidant-related targets.

Additional research was conducted through network pharmacology analysis to thoroughly investigate the intricate mechanisms through which blackberry metabolites actively participate in diverse biological processes. A total of 80 metabolites were used for the analysis: 70 with VIP scores greater than 1 from untargeted analysis and 10 from targeted analysis. These metabolites were employed to construct the compound–target (C-T) network. The results indicated that 35 metabolites formed networks with 210 genes sourced from the STITCH database (Supplementary Figure S1). The STITCH 5.0 database assigns a “combined score” to interactions between compounds and genes, with stronger interactions corresponding to higher scores. Only metabolites with interaction scores exceeding 0.700 were retained in this study (Table 2). Additionally, we constructed another C-T network to elucidate the interaction mechanism between blackberry metabolites and genes associated with inflammation and oxidative regulation. From the 210 genes, a subset related to inflammation and antioxidant properties was filtered using keywords “anti-inflammation” and “antioxidant” on GeneCards. The results revealed that 13 metabolites interacted with a network of 31 genes (Figure 6A). Among them, AKT1, CASP3, JUN, MAPK8, NOS3, NQO1, and HMOX1 were the most enriched genes. AKT1 is known to suppress the activation of immune cells and the release of proinflammatory cytokines [61]. It has also been shown to have substantial anti-inflammatory effects via suppression of NF-kB-mediated transcription. CASP3 may be involved in the process of inflammation by cleaving cytokines [62]. NQO1 and HMOX1, which play important roles in cell defense by enhancing the elimination of ROS [63]. Furthermore, NOS3 protects against systemic inflammation and myocardial dysfunction [64]. JUN is a crucial regulator of skin inflammation in the epidermis [65]. As shown in Figure 6B, the larger the degree, the stronger the relationship between the genes corresponding to the node. This indicates that the target gene, such as AKT1, MAPK8, JUN, and STAT3, plays a key role and is an important target in the whole network. These target gene were centrally located in the PPI network, indicating that these genes are involved in the process of inflammation or oxidation.

### 3.6. GO and KEGG Enrichment Analysis

**Figure 7:**
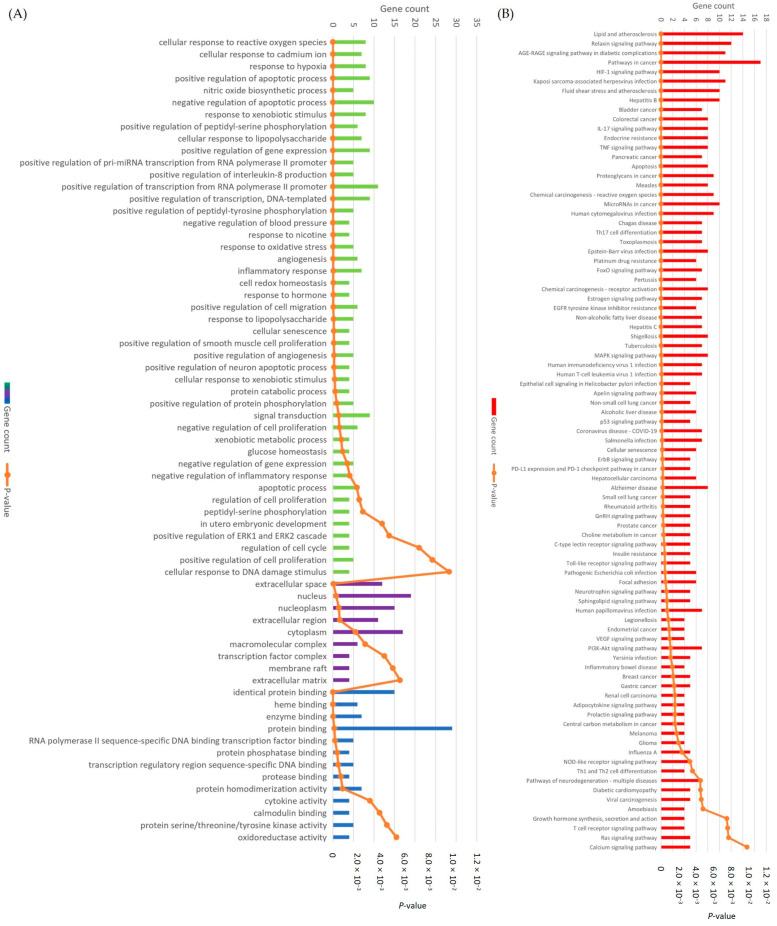
(**A**) Gene ontology enrichment analysis of anti-inflammation and antioxidant-related targets was conducted using the DAVID database. Biological processes, cellular components, and molecular functions are represented by green, purple, and blue bars, respectively. (**B**) KEGG pathway clusters generated from DAVID database. The significance of enrichment, including the *p*-value, is indicated by the orange line.

To elucidate the biological functions of 31 potential targets for 13 blackberry metabolites, the targets were characterized by GO and KEGG pathway enrichment analyses. GO enrichment analysis of target proteins that act with their corresponding ingredients was performed by DAVID. In the GO analysis, a total of 67 GO terms were found, including 45 of BP, 9 of CC, and 13 of MF (*p* < 0.01). The main GO terms of BP were related to regulation of apoptotic process, response to xenobiotic stimulus, cellular response to reactive oxygen species, cellular response to lipopolysaccharide, inflammatory response, etc. (Figure 7A). CC was mainly enriched in nucleus, cytoplasm, nucleoplasm, extracellular space, etc. MF was significantly enriched in protein binding and enzyme binding, etc. The important signaling pathways related to anti-inflammation and antioxidant were shown by KEGG pathway enrichment analysis. A total of 87 pathways were identified. The common signaling pathways mainly focused on the pathways in cancer, lipid and atherosclerosis, IL-17 signaling pathway, TNF signaling pathway, MAPK signaling pathway, PI3K-Akt signaling pathway, etc. (Figure 7B). Therefore, our findings suggest that blackberry metabolites may exert therapeutic effects by activating specific genes and modulating key pathways associated with antioxidant and anti-inflammatory responses.

## 4. Conclusions

This study compared the metabolites, antioxidant activity, and anti-inflammatory activity among six blackberry cultivars. Our correlation analysis between metabolites and biological activities enabled the prediction of influential metabolites. Notably, the “Kiowa” blackberry cultivar exhibited the highest levels of antioxidant contents and activities, as well as anti-inflammatory activities, attributed to differences in flavonoid accumulation via the phenylpropanoid pathway. Furthermore, our research utilized network pharmacology analysis to elucidate the likely mechanisms of blackberry metabolites related to anti-inflammation and antioxidant effects. This study not only deepens our understanding of the molecular mechanisms contributing to the health benefits of blackberries but also sets the stage for future research and the development of targeted interventions for conditions associated with inflammation and oxidative stress. Our findings hold the potential to influence advancements in blackberry breeding and cultivation practices, aiming to enhance their nutritional and functional qualities. However, further research is essential to fully elucidate the complicated mechanisms of these metabolites.

## Figures and Tables

**Table 2:** Potential protein targets of the blackberry constituents.

Gene ID	Protein Names	Interacting Compounds	Combined Score
ABCA1	Phospholipid-transporting ATPase ABCA1	Mevalonic acid	0.832
ABCB1	ATP-dependent translocase ABCB1	Gallic acid	0.824
ADIPOQ	Adiponectin	trans-Cinnamic acid	0.800
AHR	Aryl hydrocarbon receptor	Cyanidin-3-glucoside	0.700
AKT1	RAC-alpha serine/threonine-protein kinase	Epigallocatechin gallate	0.964
		Luteolin	0.856
ATM	Serine-protein kinase ATM	Gallic acid	0.824
CASP3	Caspase-3 subunit p12	Gallic acid	0.745
		Luteolin	0.947
CXCL2	C-X-C motif chemokine 2	Isoferulic acid	0.657
CYCS	Cytochrome c	Ferulic acid	0.819
CYP3A4	Cytochrome P450 3A4	Quercitrin	0.800
EGFR	Epidermal growth factor receptor	Luteolin	0.869
GATA3	Trans-acting T-cell-specific transcription factor GATA-3	Gallic acid	0.800
HMOX1	Heme oxygenase 1	Epicatechin	0.838
		Genipin	0.840
IL6	Interleukin-6	Epicatechin	0.800
JUN	Transcription factor Jun	Gallic acid	0.823
		Luteolin	0.946
MAPK1	Mitogen-activated protein kinase 1	Caffeic acid	0.800
MAPK8	Mitogen-activated protein kinase 8	Caffeic acid	0.818
		Epigallocatechin gallate	0.961
		Luteolin	0.951
MIF	Macrophage migration inhibitory factor	Caffeic acid	0.958
MMP1	Matrix metalloproteinase 1	Genipin	0.800
MMP2	Matrix metalloproteinase 2	Gallic acid	0.951
MMP9	Matrix metalloproteinase 9	Luteolin	0.949
NOS1	Nitric oxide synthase 1, neuronal	Epicatechin	0.813
NOS2	Nitric oxide synthase 2, inducible	Epicatechin	0.794
NOS3	Nitric oxide synthase 3, endothelial	Cyanidin-3-glucoside	0.816
		Epicatechin	0.818
		Epigallocatechin gallate	0.958
NQO1	NAD(P)H dehydrogenase [quinone] 1	Genipin	0.849
		Taxifolin	0.700
SERPINE1	Plasminogen activator inhibitor 1	Gallic acid	0.800
STAT3	Signal transducer and activator of transcription 3	Genipin	0.800
TP53	Cellular tumor antigen p53	Epigallocatechin gallate	0.958
TTR	Transthyretin	Epigallocatechin gallate	0.961
VEGFA	Vascular endothelial growth factor A	Epigallocatechin gallate	0.959

## Data Availability

All data used to support the findings of this study are included within the article.

## Supplementary Materials

The following supporting information can be downloaded at: https://www.mdpi.com/article/10.3390/antiox13030319/s1, Figure S1: Protein-protein interaction (PPI) network and compound–target network; Table S1: The information including name, formula, observed mass, calculated mass, and mass error of all annotated metabolites in six blackberry cultivars; Table S2: Optimal scan parameters for targeted compounds.

## References

[1] Takachi R., Inoue M., Ishihara J., Kurahashi N., Iwasaki M., Sasazuki S., Iso H., Tsubono Y., Tsugane S., JPHC Study Group (2008). Fruit and Vegetable Intake and Risk of Total Cancer and Cardiovascular Disease: Japan Public Health Center-Based Prospective Study. Am. J. Epidemiol..

[2] Nijveldt R.J., van Nood E., van Hoorn D.E., Boelens P.G., van Norren K., van Leeuwen P.A. (2001). Flavonoids: A Review of Probable Mechanisms of Action and Potential Applications. Am. J. Clin. Nutr..

[3] Halvorsen B.L., Carlsen M.H., Phillips K.M., Bøhn S.K., Holte K., Jacobs D.R., Blomhoff R. (2006). Content of Redox-Active Compounds (Ie, Antioxidants) in Foods Consumed in the United States. Am. J. Clin. Nutr..

[4] Kaume L., Howard L.R., Devareddy L. (2012). The Blackberry Fruit: A Review on Its Composition and Chemistry, Metabolism and Bioavailability, and Health Benefits. J. Agric. Food Chem..

[5] Strik B.C., Finn C.E., Clark J.R., Pilar Bañados M. (2008). Worldwide Production of Blackberries. Acta. Hortic..

[6] Clark J.R., Finn C.E. (2014). Blackberry Cult Ivation in the World. Rev. Bras. Frutic..

[7] Wu Y., Zhang C., Huang Z., Lyu L., Li W., Wu W. (2022). Integrative Analysis of the Metabolome and Transcriptome Provides Insights into the Mechanisms of Flavonoid Biosynthesis in Blackberry. Food Res. Int..

[8] Sariburun E., Şahin S., Demir C., Türkben C., Uylaşer V. (2010). Phenolic Content and Antioxidant Activity of Raspberry and Blackberry Cultivars. J. Food Sci..

[9] Solverson P., Albaugh G.P., Harrison D.J., Luthria D.L., Baer D.J., Novotny J.A. (2022). High-Dose Administration of Purified Cyanidin-3-Glucose or a Blackberry Extract Causes Improved Mitochondrial Function but Reduced Content in 3T3-L1 Adipocytes. Food Front..

[10] Heinonen I.M., Meyer A.S., Frankel E.N. (1998). Antioxidant Activity of Berry Phenolics on Human Low-Density Lipoprotein and Liposome Oxidation. J. Agric. Food Chem..

[11] Wang S.Y., Jiao H. (2000). Scavenging Capacity of Berry Crops on Superoxide Radicals, Hydrogen Peroxide, Hydroxyl Radicals, and Singlet Oxygen. J. Agric. Food Chem..

[12] Zhang J., Yu Q., Cheng H., Ge Y., Liu H., Ye X., Chen Y. (2018). Metabolomic Approach for the Authentication of Berry Fruit Juice by Liquid Chromatography Quadrupole Time-of-Flight Mass Spectrometry Coupled to Chemometrics. J. Agric. Food Chem..

[13] Schrimpe-Rutledge A.C., Codreanu S.G., Sherrod S.D., McLean J.A. (2016). Untargeted Metabolomics Strategies—Challenges and Emerging Directions. J. Am. Soc. Mass Spectrom..

[14] Cajka T., Fiehn O. (2016). Toward Merging Untargeted and Targeted Methods in Mass Spectrometry-Based Metabolomics and Lipidomics. Anal. Chem..

[15] Kim M.J., Lee M.Y., Shon J.C., Kwon Y.S., Liu K.-H., Lee C.H., Ku K.-M. (2019). Untargeted and Targeted Metabolomics Analyses of Blackberries–Understanding Postharvest Red Drupelet Disorder. Food Chem..

[16] Wu Y., Huang X., Yang H., Zhang S., Lyu L., Li W., Wu W. (2023). Analysis of Flavonoid-Related Metabolites in Different Tissues and Fruit Developmental Stages of Blackberry Based on Metabolome Analysis. Food Res. Int..

[17] Fan-Chiang H.-J., Wrolstad R.E. (2010). Sugar and Nonvolatile Acid Composition of Blackberries. J. AOAC Int..

[18] Makarkina M., Gruner L., Vetrova O., Matnasarova D. (2021). The Accumulation of Sugars and Organic Acids in Blackberry Fruit in the Conditions of Central Russia. BIO Web Conf..

[19] Siriwoharn T., Wrolstad R.E., Finn C.E., Pereira C.B. (2004). Influence of Cultivar, Maturity, and Sampling on Blackberry (*Rubus* L. Hybrids) Anthocyanins, Polyphenolics, and Antioxidant Properties. J. Agric. Food Chem..

[20] Mattioli R., Francioso A., Mosca L., Silva P. (2020). Anthocyanins: A Comprehensive Review of Their Chemical Properties and Health Effects on Cardiovascular and Neurodegenerative Diseases. Molecules.

[21] Poornima P., Kumar J.D., Zhao Q., Blunder M., Efferth T. (2016). Network Pharmacology of Cancer: From Understanding of Complex Interactomes to the Design of Multi-Target Specific Therapeutics from Nature. Pharmacol. Res..

[22] Tao W., Xu X., Wang X., Li B., Wang Y., Li Y., Yang L. (2013). Network Pharmacology-Based Prediction of the Active Ingredients and Potential Targets of Chinese Herbal Radix Curcumae Formula for Application to Cardiovascular Disease. J. Ethnopharmacol..

[23] Lai X., Wang X., Hu Y., Su S., Li W., Li S. (2020). Editorial: Network Pharmacology and Traditional Medicine. Front. Pharmacol..

[24] Kürbitz C., Heise D., Redmer T., Goumas F., Arlt A., Lemke J., Rimbach G., Kalthoff H., Trauzold A. (2011). Epicatechin Gallate and Catechin Gallate Are Superior to Epigallocatechin Gallate in Growth Suppression and Anti-Inflammatory Activities in Pancreatic Tumor Cells. Cancer Sci..

[25] Huang C.-C., Fang J.-Y., Wu W.-B., Chiang H.-S., Wei Y.-J., Hung C.-F. (2005). Protective Effects of (−)-Epicatechin-3-Gallate on UVA-Induced Damage in HaCaT Keratinocytes. Arch. Dermatol. Res..

[26] Tang J., Diao P., Shu X., Li L., Xiong L. (2019). Quercetin and Quercitrin Attenuates the Inflammatory Response and Oxidative Stress in LPS-Induced RAW264.7 Cells: In Vitro Assessment and a Theoretical Model. BioMed Res. Int..

[27] Wang Z., Gmitter F.G., Grosser J.W., Wang Y. (2022). Natural Sweeteners and Sweetness-Enhancing Compounds Identified in Citrus Using an Efficient Metabolomics-Based Screening Strategy. J. Agric. Food Chem..

[28] Suh D.H., Jung E.S., Lee G.M., Lee C.H. (2018). Distinguishing Six Edible Berries Based on Metabolic Pathway and Bioactivity Correlations by Non-Targeted Metabolite Profiling. Front. Plant Sci..

[29] Sung J., Lee J. (2010). Antioxidant and Antiproliferative Activities of Grape Seeds from Different Cultivars. Food Sci. Biotechnol..

[30] Lee H., Lee J. (2021). Anti-Diabetic Effect of Hydroxybenzoic Acid Derivatives in Free Fatty Acid-Induced HepG2 Cells via miR-1271/IRS1/PI3K/AKT/FOXO1 Pathway. J. Food Biochem..

[31] Cebulak T., Oszmiański J., Kapusta I., Lachowicz S. (2019). Effect of Abiotic Stress Factors on Polyphenolic Content in the Skin and Flesh of Pear by UPLC-PDA-Q/TOF-MS. Eur. Food Res. Technol..

[32] Chen X., Cai W., Xia J., Yu H., Wang Q., Pang F., Zhao M. (2020). Metabolomic and Transcriptomic Analyses Reveal That Blue Light Promotes Chlorogenic Acid Synthesis in Strawberry. J. Agric. Food Chem..

[33] Fayek N.M., Farag M.A., Saber F.R. (2021). Metabolome Classification via GC/MS and UHPLC/MS of Olive Fruit Varieties Grown in Egypt Reveal Pickling Process Impact on Their Composition. Food Chem..

[34] Veberic R., Stampar F., Schmitzer V., Cunja V., Zupan A., Koron D., Mikulic-Petkovsek M. (2014). Changes in the contents of anthocyanins and other compounds in blackberry fruits due to freezing and long-term frozen storage. J. Agric. Food Chem..

[35] Robinson J.A., Bierwirth J.E., Greenspan P., Pegg R.B. (2020). Blackberry Polyphenols: Review of Composition, Quantity, and Health Impacts from in Vitro and in Vivo Studies. J. Food Bioact..

[36] Schulz M., Seraglio S.K.T., Della Betta F., Nehring P., Valese A.C., Daguer H., Gonzaga L.V., Costa A.C.O., Fett R. (2019). Blackberry (*Rubus ulmifolius* Schott): Chemical Composition, Phenolic Compounds and Antioxidant Capacity in Two Edible Stages. Food Res. Int..

[37] da Rosa C.G., Borges C.D., Zambiazi R.C., Rutz J.K., da Luz S.R., Krumreich F.D., Benvenutti E.V., Nunes M.R. (2014). Encapsulation of the Phenolic Compounds of the Blackberry (*Rubus fruticosus*). LWT—Food Sci. Technol..

[38] Sellappan S., Akoh C.C., Krewer G. (2002). Phenolic Compounds and Antioxidant Capacity of Georgia-Grown Blueberries and Blackberries. J. Agric. Food Chem..

[39] Hassimotto N.M.A., da Mota R.V., Cordenunsi B.R., Lajolo F.M. (2008). Physico-Chemical Characterization and Bioactive Compounds of Blackberry Fruits (*Rubus* Sp.) Grown in Brazil. Food Sci. Technol..

[40] Pantelidis G.E., Vasilakakis M., Manganaris G.A., Diamantidis G. (2007). Antioxidant Capacity, Phenol, Anthocyanin and Ascorbic Acid Contents in Raspberries, Blackberries, Red Currants, Gooseberries and Cornelian Cherries. Food Chem..

[41] Wang S.Y., Lin H.S. (2000). Antioxidant Activity in Fruits and Leaves of Blackberry, Raspberry, and Strawberry Varies with Cultivar and Developmental Stage. J. Agric. Food Chem..

[42] Cuevas-Rodríguez E.O., Yousef G.G., García-Saucedo P.A., López-Medina J., Paredes-López O., Lila M.A. (2010). Characterization of Anthocyanins and Proanthocyanidins in Wild and Domesticated Mexican Blackberries (*Rubus* Spp.). J. Agric. Food Chem..

[43] Kim J.-S. (2018). Antioxidant Activities of Selected Berries and Their Free, Esterified, and Insoluble-Bound Phenolic Acid Contents. Prev. Nutr. Food Sci..

[44] Krzepiłko A., Prażak R., Święciło A. (2021). Chemical Composition, Antioxidant and Antimicrobial Activity of Raspberry, Blackberry and Raspberry-Blackberry Hybrid Leaf Buds. Molecules.

[45] Forrester S.J., Kikuchi D.S., Hernandes M.S., Xu Q., Griendling K.K. (2018). Reactive Oxygen Species in Metabolic and Inflammatory Signaling. Circ. Res..

[46] Roy A., Das S., Chatterjee I., Roy S., Chakraborty R., Ekiert H.M., Ramawat K.G., Arora J. (2022). Anti-Inflammatory Effects of Different Dietary Antioxidants. Plant Antioxidants and Health.

[47] Wu L., Ashraf M.H.N., Facci M., Wang R., Paterson P.G., Ferrie A., Juurlink B.H.J. (2004). Dietary Approach to Attenuate Oxidative Stress, Hypertension, and Inflammation in the Cardiovascular System. Proc. Natl. Acad. Sci. USA.

[48] Ma Z., Du B., Li J., Yang Y., Zhu F. (2021). An Insight into Anti-Inflammatory Activities and Inflammation Related Diseases of Anthocyanins: A Review of Both In Vivo and In Vitro Investigations. Int. J. Mol. Sci..

[49] Wongwichai T., Teeyakasem P., Pruksakorn D., Kongtawelert P., Pothacharoen P. (2019). Anthocyanins and Metabolites from Purple Rice Inhibit IL-1β-Induced Matrix Metalloproteinases Expression in Human Articular Chondrocytes through the NF-κB and ERK/MAPK Pathway. Biomed. Pharmacother..

[50] Bernatova I., Liskova S. (2021). Mechanisms Modified by (−)-Epicatechin and Taxifolin Relevant for the Treatment of Hypertension and Viral Infection: Knowledge from Preclinical Studies. Antioxidants.

[51] Aziz N., Kim M.-Y., Cho J.Y. (2018). Anti-Inflammatory Effects of Luteolin: A Review of in Vitro, in Vivo, and in Silico Studies. J. Ethnopharmacol..

[52] Zhao D., Liu K., Wang J., Shao H. (2023). Syringin Exerts Anti-Inflammatory and Antioxidant Effects by Regulating SIRT1 Signaling in Rat and Cell Models of Acute Myocardial Infarction. Immun. Inflamm. Dis..

[53] Ginting C., Lister I.N., Girsang E., Mutia M., Lubis Y., Amalia A., Rizal R., Widowati W. (2019). Anti-Inflammatory Activity of Quercitrin on Hypoxia-Induced EA.Hy926. J. Phys. Conf. Ser..

[54] Li X., Jiang Q., Wang T., Liu J., Chen D. (2016). Comparison of the Antioxidant Effects of Quercitrin and Isoquercitrin: Understanding the Role of the 6″-OH Group. Molecules.

[55] Kotanidou A., Xagorari A., Bagli E., Kitsanta P., Fotsis T., Papapetropoulos A., Roussos C. (2002). Luteolin Reduces Lipopolysaccharide-Induced Lethal Toxicity and Expression of Proinflammatory Molecules in Mice. Am. J. Respir. Crit. Care Med..

[56] Chen X., Kitts D. (2017). Demonstrating the Relationship between the Phytochemical Profile of Different Teas with Relative Antioxidant and Anti-Inflammatory Capacities. Funct. Foods Health Dis..

[57] Cheynier V., Comte G., Davies K.M., Lattanzio V., Martens S. (2013). Plant Phenolics: Recent Advances on Their Biosynthesis, Genetics, and Ecophysiology. Plant Physiol. Biochem..

[58] Seong E., Heo H., Sang Jeong H., Lee H., Lee J. (2023). Enhancement of Bioactive Compounds and Biological Activities of Centella Asiatica through Ultrasound Treatment. Ultrason. Sonochem..

[59] Yu J., Lee H., Heo H., Jeong H.S., Sung J., Lee J. (2023). Sucrose-Induced Abiotic Stress Improves the Phytochemical Profiles and Bioactivities of Mung Bean Sprouts. Food Chem..

[60] Sim U., Sung J., Lee H., Heo H., Jeong H.S., Lee J. (2020). Effect of Calcium Chloride and Sucrose on the Composition of Bioactive Compounds and Antioxidant Activities in Buckwheat Sprouts. Food Chem..

[61] He X., Li Y., Deng B., Lin A., Zhang G., Ma M., Wang Y., Yang Y., Kang X. (2022). The PI3K/AKT Signalling Pathway in Inflammation, Cell Death and Glial Scar Formation after Traumatic Spinal Cord Injury: Mechanisms and Therapeutic Opportunities. Cell Prolif..

[62] Wu D., Wang Z., Zhang J., Robinson A.G., Lyu B., Chen Z., Wang C., Wei B., Xia X., Zhang Q. (2022). Apoptotic Caspase Inhibits Innate Immune Signaling by Cleaving NF-κBs in Both Mammals and Flies. Cell Death Dis..

[63] Yu M., Kim H.J., Heo H., Kim M., Jeon Y., Lee H., Lee J. (2022). Comparison of the Antihypertensive Activity of Phenolic Acids. Molecules.

[64] Bougaki M., Searles R.J., Kida K., Yu J., Buys E.S., Ichinose F. (2010). Nos3 Protects against Systemic Inflammation and Myocardial Dysfunction in Murine Polymicrobial Sepsis. Shock.

[65] Schonthaler H.B., Guinea-Viniegra J., Wagner E.F. (2011). Targeting Inflammation by Modulating the Jun/AP-1 Pathway. Ann. Rheum. Dis..

