# Supplementary material for: Assessment of Six Blackberry Cultivars Using a Combination of Metabolomics, Biological Activity, and Network Pharmacology Approaches
Source: Antioxidants (Basel). 2024 Mar 6;13(3):319. doi: 10.3390/antiox13030319 (PMC10967939; doi:10.3390/antiox13030319)
Supplement: Supplementary file 1 [file antioxidants-13-00319-s001.zip › antioxidants-2889166-supplementary.pdf]

Supplementary Materials

Assessment of six blackberry cultivars using a combination of metabolomics, biological activity, and network pharmacology approaches

Hana Lee [1], Zhixin Wang [1], Zhanao Deng [2] and Yu Wang [1,*]

[1] Department of Food Science and Human Nutrition, Citrus Research and Education Center, University of Florida, Lake Alfred, Florida, 33850, USA; lee.hana@ufl.edu, zhixin.wang@ufl.edu

[2] Department of Environmental Horticulture, Gulf Coast Research and Education Center, University of Florida, IFAS, Wimauma, Florida, 33598, USA; zdeng@ufl.edu

* Correspondence: yu.wang@ufl.edu; Tel.: +1-863-956-8673

Table S1. The information of all annotated metabolites in six blackberry cultivars.

Name	Formula	Observed mass	Calculated mass	Mass error (ppm)
(+)-Camphor	C10 H16 O	152.12012	152.11983	-1.89
(2S,3S)-2-Hydroxytridecane-1,2,3-tricarboxylic acid	C16 H28 O7	332.1835	332.18335	-0.45
(R)-(Homo)2-citrate	C8 H12 O7	220.0583	220.05803	-1.22
(R)-2-Hydroxyglutarate	C5 H8 O5	148.03717	148.03705	-0.81
(S)-1-Piperideine-6-carboxylic acid	C6 H9 N O2	127.06333	127.06302	-2.39
(S)-2-(Hydroxymethyl)glutarate	C6 H10 O5	162.05282	162.05272	-0.65
(S)-2-Hydroxyglutarate	C5 H8 O5	148.03717	148.03702	-1.01
1,2,3-Trihydroxybenzene	C6 H6 O3	126.03169	126.03138	-2.52
1,2-Benzoquinone	C6 H4 O2	108.02113	108.02094	-1.71
1-Galloyl-beta-glucose	C13 H16 O10	332.07435	332.07423	-0.36
1-O-Vanilloyl-beta-D-glucose	C14 H18 O9	330.09508	330.09488	-0.6
1-Pyrroline-2-carboxylic acid	C5 H7 N O2	113.04768	113.04744	-2.1
2-(4-Ethoxyphenyl)-5,6,7,8-tetramethoxy-4H-1-benzopyran-4-one	C21 H22 O7	386.13655	386.13556	-2.56
2,3-Dihydroxy-3-methylbutanoate	C5 H10 O4	134.05791	134.05781	-0.73
2,3-Dihydroxy-p-cumate	C10 H12 O4	196.07356	196.07308	-2.44
2,3-Dimethylmaleate	C6 H8 O4	144.04226	144.04191	-2.39
2,4-Dihydroxyhept-2-enedioate	C7 H10 O6	190.04774	190.04765	-0.47
2-Amino-3,7-dideoxy-D-threo-hept-6-ulosonic acid	C7 H13 N O5	191.07937	191.07891	-2.42
2-Aminobenzoic acid	C7 H7 N O2	137.04768	137.04729	-2.81
2-Butyloctyl sulfate	C12 H26 O4 S	266.15518	266.15501	-0.63
2-Dehydro-3-deoxy-D-fuconate	C6 H10 O5	162.05282	162.05266	-1.04
2-Dehydro-3-deoxy-L-arabinonate	C5 H8 O5	148.03717	148.0371	-0.53
2-Formyl-1-indanone	C10 H8 O2	160.05243	160.05204	-2.43
2-Hydroxy-2,4-pentadienoate	C5 H6 O3	114.03169	114.03143	-2.3
2-Hydroxy-3-oxoadipate	C6 H8 O6	176.03209	176.03187	-1.24
2-Hydroxy-5-methylquinone	C7 H6 O3	138.03169	138.03145	-1.78
2-Hydroxyadipate	C6 H10 O5	162.05282	162.05267	-0.93
2-Hydroxyglutaric acid	C5 H8 O5	148.03717	148.03713	-0.29
2-Hydroxymethyl succinate semialdehyde	C5 H8 O4	132.04226	132.0421	-1.19
2-Hydroxymethylclavam	C6 H9 N O3	143.05824	143.05798	-1.85
2-Hydroxyphenylpropanoate	C9 H10 O3	166.06299	166.06258	-2.51
2-Maleylacetate	C6 H6 O5	158.02152	158.02136	-1.03
2-O-(alpha-D-Mannosyl)-D-glycerate	C9 H16 O9	268.07943	268.0792	-0.86
2-Oxoadipate	C6 H8 O5	160.03717	160.03694	-1.43
2-Oxopimelate	C7 H10 O5	174.05282	174.05258	-1.38
2-Oxosuberate	C8 H12 O5	188.06847	188.06826	-1.14
2-Phenylethanol	C8 H10 O	122.07316	122.07286	-2.51

2-Propylglutaric acid	C8 H14 O4	174.08921	174.08903	-1.01
3,4-Dihydroxystyrene	C8 H8 O2	136.05243	136.05219	-1.75
3-Deoxy-lyxo-heptulosaric acid	C7 H10 O8	222.03757	222.03738	-0.83
3-Methyleneoxindole	C9 H7 N O	145.05276	145.05248	-1.96
3-Propylmalate	C7 H12 O5	176.06847	176.06829	-1.06
4-Acetamidobutanoic acid	C6 H11 N O3	145.07389	145.07362	-1.88
4-Guanidinobutyric acid	C5 H11 N3 O2	145.08513	145.08484	-1.96
4-Hydroxy-2-oxoglutaric acid	C5 H6 O6	162.01644	162.01631	-0.76
4-Hydroxybenzoate-O-glucoside	C13 H16 O8	300.08452	300.0842	-1.04
4-Hydroxybenzoic acid	C7 H6 O3	138.03169	138.03163	-0.43
4-Hydroxymuconic semialdehyde	C6 H6 O4	142.02661	142.02651	-0.69
4-Hydroxyphenyllactic acid	C9 H10 O4	182.05791	182.05749	-2.29
4-Methyl-3-oxoadipate	C7 H10 O5	174.05282	174.05255	-1.54
4-Methylene-L-glutamate	C6 H9 N O4	159.05316	159.05296	-1.22
4-O-Methyl-D-glucuronic acid	C7 H12 O7	208.0583	208.05817	-0.65
4-Oxoproline	C5 H7 N O3	129.04259	129.04234	-1.95
4-Propylphenol	C9 H12 O	136.08881	136.08851	-2.22
5-(2'-Carboxyethyl)-4,6-dihydroxypicolinate	C9 H9 N O6	227.04299	227.04253	-1.99
5-Acetamidovaleric acid	C7 H13 N O3	159.08954	159.08918	-2.28
5-Methyl-2-furaldehyde	C6 H6 O2	110.03678	110.0365	-2.52
5-Oxo-1,2-campholide	C10 H14 O3	182.09429	182.09386	-2.4
5-Oxohexanoic acid	C6 H10 O3	130.06299	130.06302	0.2
6-Acetyl-D-glucose	C8 H14 O7	222.07395	222.07376	-0.85
6-Methylsalicylate	C8 H8 O3	152.04734	152.04717	-1.14
7,8-Dihydrobiopterin	C9 H13 N5 O3	239.10184	239.10127	-2.4
7-Deoxyloganetin	C11 H16 O4	212.10486	212.10426	-2.82
7-Methoxycoumarin	C10 H8 O3	176.04734	176.04688	-2.62
Acanthoside B	C28 H36 O13	580.21559	580.21516	-0.74
Acetylpyruvate	C5 H6 O4	130.02661	130.02652	-0.71
alpha-Methylstyrene	C9 H10	118.07825	118.07801	-2.04
Aromatic aldehyde	C7 H6 O	106.04186	106.04159	-2.62
Azelaic acid	C9 H16 O4	188.10486	188.10474	-0.63
Benzaldehyde	C7 H6 O	106.04186	106.04159	-2.55
Benzofuran	C8 H6 O	118.04186	118.04162	-2.08
Benzoylformic acid	C8 H6 O3	150.03169	150.03136	-2.24
Benzyl 2-methyl-3-oxobutanoate	C12 H14 O3	206.09429	206.09373	-2.73
Benzyl alcohol	C7 H8 O	108.05751	108.0574	-1.09
beta-Cymaropyranose	C7 H14 O4	162.08921	162.08911	-0.61
Caffeic acid	C9 H8 O4	180.04226	180.04173	-2.91

Caffeyl alcohol	C9 H10 O3	166.06299	166.0626	-2.39
Calystegin B2	C7 H13 N O4	175.08446	175.08409	-2.1
Carpacin	C11 H12 O3	192.07864	192.07825	-2.06
Catechol	C6 H6 O2	110.03678	110.03651	-2.45
Cellobionate	C12 H22 O12	358.11113	358.11101	-0.33
Cinnamaldehyde	C9 H8 O	132.05751	132.05721	-2.34
cis-1,2-Dihydronaphthalene-1,2-diol	C10 H10 O2	162.06808	162.06772	-2.23
cis-2,3-Dihydro-2,3-dihydroxybiphenyl	C12 H12 O2	188.08373	188.08319	-2.89
cis-Aconitic acid	C6 H6 O6	174.01644	174.01615	-1.64
cis-Homoaconitate	C7 H8 O6	188.03209	188.0319	-1.02
Citric acid	C6 H8 O7	192.027	192.02673	-1.43
Coumarin	C9 H6 O2	146.03678	146.03648	-2.05
Cyclohexane-1,3-dione	C6 H8 O2	112.05243	112.05211	-2.86
Cyclohexylamine	C6 H13 N	99.1048	99.10452	-2.84
Cytosine	C4 H5 N3 O	111.04326	111.04296	-2.71
Decanedioic acid	C10 H18 O4	202.12051	202.12047	-0.18
Deidaclin	C12 H17 N O6	271.10559	271.10506	-1.94
D-Fructose 6-phosphate	C6 H13 O9 P	260.02972	260.02942	-1.15
D-Gluconic acid	C6 H12 O7	196.0583	196.05809	-1.06
D-Glucose	C6 H12 O6	180.06339	180.0632	-1.04
D-Glucose 6-phosphate	C6 H13 O9 P	260.02972	260.02939	-1.25
D-Glycerate	C3 H6 O4	106.02661	106.02647	-1.31
Difructose anhydride	C12 H20 O10	324.10565	324.105	-1.98
Digitalose	C7 H14 O5	178.08412	178.08397	-0.84
Dihydrocaffeic acid	C9 H10 O4	182.05791	182.0578	-0.57
D-Leucate	C6 H12 O3	132.07864	132.07857	-0.57
Dodecanedioic acid	C12 H22 O4	230.15181	230.15164	-0.73
D-Xylonate	C5 H10 O6	166.04774	166.04745	-1.74
Ethyl cinnamate	C11 H12 O2	176.08373	176.08332	-2.31
Ferulic acid	C10 H10 O4	194.05791	194.05774	-0.89
gamma-Aminobutyric acid	C4 H9 N O2	103.06333	103.06306	-2.62
Genipin	C11 H14 O5	226.08412	226.08354	-2.58
Glucovanillin	C14 H18 O8	314.10017	314.10006	-0.33
Glucuronic acid	C6 H10 O7	194.04265	194.04238	-1.4
Glucuronolactone	C6 H8 O6	176.03209	176.03168	-2.3
Glutamyl-glutamic acid	C10 H16 N2 O7	276.09575	276.09571	-0.15
Glutarate	C5 H8 O4	132.04226	132.04211	-1.14
Glycyl-leucine	C8 H16 N2 O3	188.11609	188.1157	-2.1
Homogentisic acid	C8 H8 O4	168.04226	168.04179	-2.77

Indan-1-ol	C9 H10 O	134.07316	134.0728	-2.69
Isoferulic acid	C10 H10 O4	194.05791	194.05771	-1.03
Jasmone	C11 H16 O	164.12012	164.11979	-2
Ketodeoxyoctonate	C8 H14 O8	238.06887	238.06871	-0.67
L-2-Aminoadipate	C6 H11 N O4	161.06881	161.06844	-2.25
Lariciresinol	C20 H24 O6	360.15729	360.15634	-2.63
L-Ascorbic acid-2-glucoside	C12 H18 O11	338.08491	338.08484	-0.2
Lentiginosine	C8 H15 N O2	157.11028	157.10996	-2.05
L-Formylkynurenine	C11 H12 N2 O4	236.07971	236.07932	-1.66
L-Glutamate	C5 H9 N O4	147.05316	147.05301	-1.04
L-Glutamate 5-semialdehyde	C5 H9 N O3	131.05824	131.0581	-1.08
Limonene	C10 H16	136.1252	136.12497	-1.69
L-Pipecolate	C6 H11 N O2	129.07898	129.07875	-1.78
L-Serine	C3 H7 N O3	105.04259	105.04235	-2.35
L-Tyrosine	C9 H11 N O3	181.07389	181.07342	-2.64
L-Tyrosine methyl ester	C10 H13 N O3	195.08954	195.08914	-2.06
Luteolin	C15 H10 O6	286.04774	286.04691	-2.88
L-Valine	C5 H11 N O2	117.07898	117.07871	-2.33
Malic acid	C4 H6 O5	134.02152	134.02143	-0.72
Melibiitol	C12 H24 O11	344.13186	344.13161	-0.73
Methyl beta-D-galactoside	C7 H14 O6	194.07904	194.07878	-1.34
Mevalonic acid	C6 H12 O4	148.07356	148.07346	-0.64
N-(3-Oxohexanoyl)homoserine lactone	C10 H15 N O4	213.10011	213.09964	-2.21
N,N-Dihydroxy-L-valine	C5 H11 N O4	149.06881	149.06851	-2.01
N2-Acetyl-L-aminoadipate	C8 H13 N O5	203.07937	203.07928	-0.45
N-Acetyl-beta-D-glucosaminylamine	C8 H16 N2 O5	220.10592	220.10552	-1.84
N-Acetyl-D-glucosaminate	C8 H15 N O7	237.08485	237.08471	-0.59
N-Acetyl-L-2-amino-6-oxopimelate	C9 H13 N O6	231.07429	231.07405	-1.01
N-Acetyl-L-phenylalanine	C11 H13 N O3	207.08954	207.08927	-1.31
N-Acetylornithine	C7 H14 N2 O3	174.10044	174.10006	-2.21
N-Butyryl-L-homoserine lactone	C8 H13 N O3	171.08954	171.08915	-2.28
N-Ethylmaleimide	C6 H7 N O2	125.04768	125.04742	-2.04
N-Hydroxy-L-valine	C5 H11 N O3	133.07389	133.07361	-2.09
Nicotianamine	C12 H21 N3 O6	303.14304	303.14256	-1.56
Nicotinamide	C6 H6 N2 O	122.04801	122.04776	-2.05
Nicotinic acid	C6 H5 N O2	123.03203	123.0318	-1.86
Octylamine	C8 H19 N	129.15175	129.15156	-1.44
Oxoglutaric acid	C7 H8 O7	204.027	204.02687	-0.64
Pantetheine	C11 H22 N2 O4 S	278.13003	278.12974	-1.04

Pantothenate	C9 H17 N O5	219.11067	219.11042	-1.15
p-Coumaraldehyde	C9 H8 O2	148.05243	148.05213	-2.05
p-Coumaric acid	C9 H8 O3	164.04734	164.04728	-0.36
Phenyllactylglucose	C15 H20 O8	328.11582	328.11572	-0.29
Picolinamide	C6 H6 N2 O	122.04801	122.04773	-2.36
Plantagoside	C21 H22 O12	466.11113	466.11063	-1.06
Proclavaminic acid	C8 H14 N2 O4	202.09536	202.09489	-2.32
Prolylleucine	C11 H20 N2 O3	228.14739	228.147	-1.74
Pyridoxine	C8 H11 N O3	169.07389	169.07345	-2.61
Quercitrin	C21 H20 O11	448.10056	448.09906	-3.36
Quinic acid	C7 H12 O6	192.06339	192.06326	-0.65
Secologanate	C16 H22 O10	374.1213	374.12119	-0.27
Sinapyl alcohol	C11 H14 O4	210.08921	210.08861	-2.86
Succinate semialdehyde	C4 H6 O3	102.03169	102.03155	-1.44
Syringin	C17 H24 O9	372.14203	372.14172	-0.85
Taxifolin	C15 H12 O7	304.0583	304.05769	-2.01
Threonate	C4 H8 O5	136.03717	136.03713	-0.33
trans-Cinnamic acid	C9 H8 O2	148.05243	148.05227	-1.05
Triacetate	C6 H8 O4	144.04226	144.04208	-1.23
Tuberonic acid glucoside	C18 H28 O9	388.17333	388.17329	-0.11
Uridine 5'-diphosphate	C9 H14 N2 O12 P2	404.0022	404.00145	-1.85
Vanillic acid	C8 H8 O4	168.04226	168.04208	-1.07
Xylitol	C5 H12 O5	152.06847	152.06834	-0.87
Xylobiose	C10 H18 O9	282.09508	282.09488	-0.73

Table S2. Optimal scan parameters for targeted compounds

Compound	RT (min)	Polarity	Precursor (m/z)	Product (m/z)	Collison energy (V)	RF lens (V)
Cyanidin-3-glucoside	2.65	+	449.1	287.1	23	229
Cyanidin-3-rutinoside	2.64	+	595.2	287.1	30	249
Epicatechin	3.64	+	291.1	139.1	17	63
Epicatechin gallate	4.70	+	443.1	273.1	10	163
Epigallocatechin gallate	3.66	+	459.1	289.1	10	192
Genistein-d_4	7.45	+	275.1	219.1	25	229
Gallic acid	1.70	–	168.8	125.1	16	175
Vanillic acid	2.20	–	167	152	15	126
Coumaric acid	2.00	–	163.1	119.1	16	135
Ferulic acid	1.90	–	193	134.1	17	143
Sinapic acid	1.91	–	223.1	208	14	166
Salicylic acid-d_4	1.78	–	141	97.1	19	122

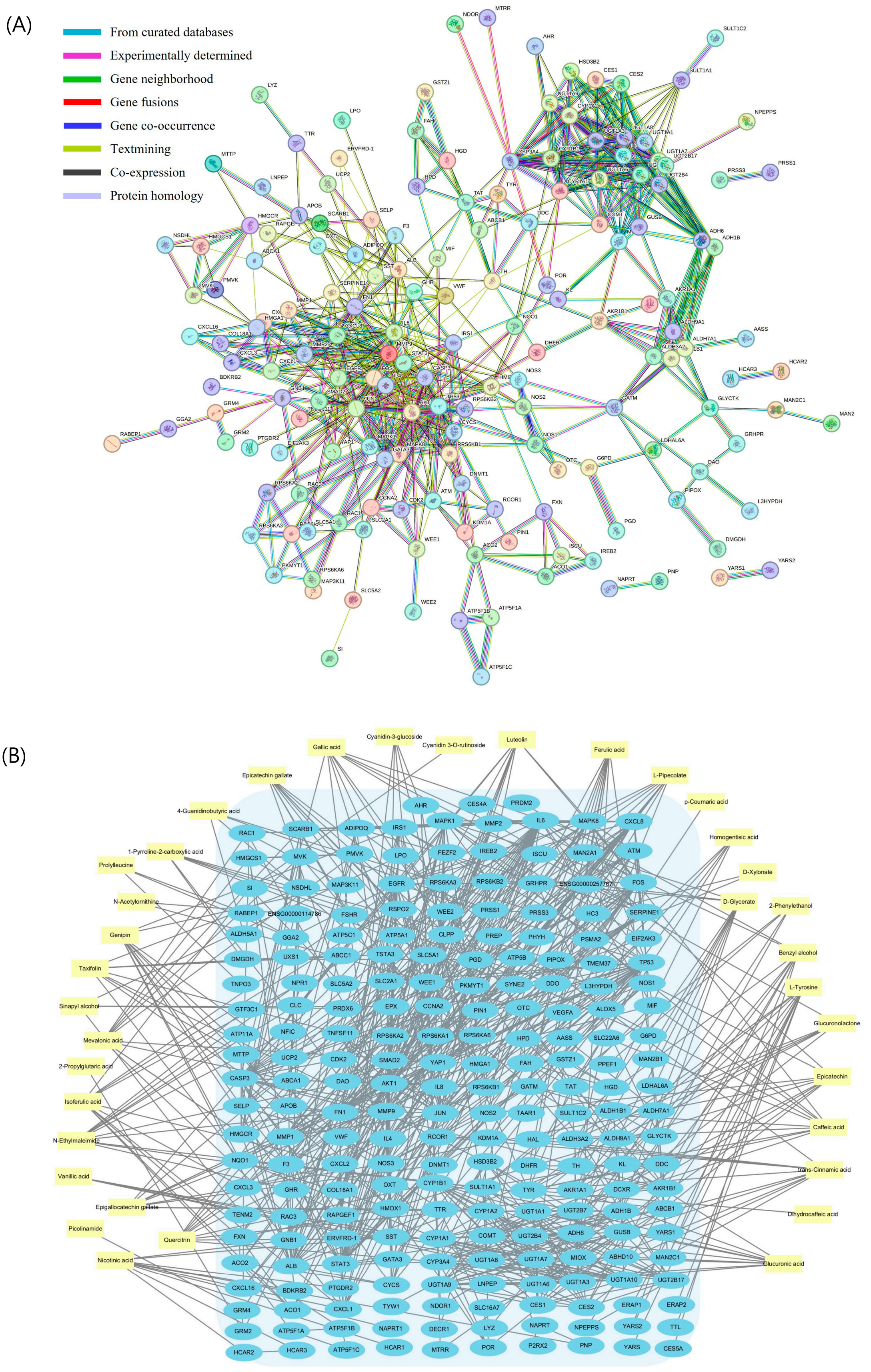

Figure S1. (A) Protein-protein interaction (PPI) network and (B) Compound-target network.
